# Overdiagnosis in the population-based organized breast cancer screening program estimated by a non-homogeneous multi-state model: a cohort study using individual data with long-term follow-up

**DOI:** 10.1186/s13058-018-1082-z

**Published:** 2018-12-17

**Authors:** Wendy Yi-Ying Wu, Sven Törnberg, Klara Miriam Elfström, Xijia Liu, Lennarth Nyström, Håkan Jonsson

**Affiliations:** 1 Department of Radiation Sciences, Oncology, Umeå University, 90187 Umeå, Sweden; 2 Department of Oncology-Pathology, Karolinska Institute, 17177 Solna, Stockholm, Sweden; 3 Regional Cancer Center Stockholm-Gotland, 11827 Stockholm, Sweden; 4 Department of Mathematics and Mathematical Statistics, Umeå University, 90187 Umeå, Sweden; 5 Department of Public Health and Clinical Medicine, Epidemiology and Global Health, Umeå University, 90187 Umeå, Sweden

**Keywords:** Overdiagnosis, Breast cancer, Organized screening program, Mammography, Multi-state model

## Abstract

**Background:**

Overdiagnosis, defined as the detection of a cancer that would not become clinically apparent in a woman’s lifetime without screening, has become a growing concern. Similar underlying risk of breast cancer in the screened and control groups is a prerequisite for unbiased estimates of overdiagnosis, but a contemporary control group is usually not available in organized screening programs.

**Methods:**

We estimated the frequency of overdiagnosis of breast cancer due to screening in women 50–69 years old by using individual screening data from the population-based organized screening program in Stockholm County 1989–2014. A hidden Markov model with four latent states and three observed states was constructed to estimate the natural progression of breast cancer and the test sensitivity. Piecewise transition rates were used to consider the time-varying transition rates. The expected number of detected non-progressive breast cancer cases was calculated.

**Results:**

During the study period, 2,333,153 invitations were sent out; on average, the participation rate in the screening program was 72.7% and the average recall rate was 2.48%. In total, 14,648 invasive breast cancer cases were diagnosed; among the 8305 screen-detected cases, the expected number of non-progressive breast cancer cases was 35.9, which is equivalent to 0.43% (95% confidence interval (CI) 0.10%–2.2%) overdiagnosis. The corresponding estimates for the prevalent and subsequent rounds were 15.6 (0.87%, 95% CI 0.20%–4.3%) and 20.3 (0.31%, 95% CI 0.07%–1.6%), respectively. The likelihood ratio test showed that the non-homogeneous model fitted the data better than an age-homogeneous model (*P* <0.001).

**Conclusions:**

Our findings suggest that overdiagnosis in the organized biennial mammographic screening for women 50–69 in Stockholm County is a minor phenomenon. The frequency of overdiagnosis in the prevalent screening round was higher than that in subsequent rounds. The non-homogeneous model performed better than the simpler, traditional homogeneous model.

**Electronic supplementary material:**

The online version of this article (10.1186/s13058-018-1082-z) contains supplementary material, which is available to authorized users.

## Background

The population-based organized screening program with mammography in Stockholm County started in 1989. During the first two years (1989–1990), 80,000 women per year were invited; during the latest five years (2010–2014), the number of women invited per year has increased to 100,000. In a meta-analysis in which 13 areas within nine counties in Sweden were combined, a 43% mortality reduction was found for women actually screened in the screening epoch compared with the pre-screening epoch after adjustment for self-selection bias. In the four areas in Stockholm County, the mortality reductions were 36%–54% and 18%–41% in women screened and invited, respectively [1].

Harms of screening, especially overdiagnosis, have become a growing concern. Overdiagnosis is defined as the detection of a cancer that would not become clinically apparent in a woman’s lifetime without screening. It can result from either detecting a non-progressive cancer or detecting a progressive cancer and the patient dies before the cancer becomes clinically detectable. However, on the individual level, it is currently impossible to determine whether a screen-detected cancer has been overdiagnosed or not. The frequency of overdiagnosis can be estimated at only a group level, which complicates estimation.

Ideally, the frequency of overdiagnosis can be estimated from comparing the excess cumulative breast cancer (BC) incidence between screened and unscreened women. Similar underlying risk of BC in the two groups is a prerequisite for unbiased estimates which might be the case in randomized controlled trials (RCTs), but a contemporary control group is usually not available in the organized screening since the entire population is invited [2]. A possibility is to estimate the incidence rate in the pre-screening period and then extrapolate to obtain an expected incidence in the absence of screening. However, the incidence trend in the screening period in the absence of screening is unknown and assumptions made in extrapolating the incidence rate—such as type of regression model, duration of pre-screening period, and screened age range—will have an impact on the estimates [3]. An alternative is to estimate the frequency of overdiagnosis using multi-state models through estimating the natural history of BC during the screening epoch only.

Multi-state models have been widely used to characterize the natural course of diseases. The simplest model describing the progression of BC includes three states: free of BC, preclinical screen-detectable phase (PCDP) (asymptomatic but detectable by screening), and clinical phase (CP) (disease with clinical symptoms). The rate of moving to another state is called the transition rate, and the duration of staying in the PCDP is called the sojourn time. Subjects with long sojourn times can be thought of as slow-growing or non-progressive cancers that may be overdiagnosed cases if detected by screening. The three-state model can be extended to a four-state model by dividing the preclinical phase into progressive and non-progressive PCDPs to represent the true early detected cases and overdiagnosed cases if they were detected by screening. Several multi-state models using constant rate have been developed to estimate the frequency of overdiagnosis in BC screening [4–7]. In this study, we developed a non-homogeneous model to cope with age-specific transition rates. In a recent study, the estimates from this model were validated and found to be comparable to the results from the cumulative incidence approach in a randomized trial setting [8]. We applied this method to estimate the frequency of overdiagnosis in the organized screening program in Stockholm County, Sweden, using individual data on screening history and mode of detection.

## Methods

### Study population

Population-based, organized screening with mammography started in Stockholm in 1989. Women 50–69 years old were invited to screening every 24 months. Between 2005 and 2009, the screening program was gradually extended to women 40–49 years old; from 2012, women 70–74 years old were also invited. To estimate the frequency of overdiagnosis for women 50–69 years old, women born in 1920–59 and invited to screening in 1989–2014 (*N* = 417,710) were considered; that is, women 40–49 years old at invitation were not included in the study. Only 9.65% of women born in 1938–44 were invited to screening after age 69 (*N* = 40,308).

From the start of screening, individual screening information on invitation, participation, recall for further assessment, and screening results as well as findings from the diagnostic procedures following a positive screening result were regularly recorded in the regional screening register [9]. The unique identification number for each woman was used to link the screening data to the Stockholm-Gotland Cancer Register (which is a regional part of the national cancer register founded in 1958) to identify BC cases. International Classification of Disease (ICD) site code 170 or C50 and histo-pathological code C − 24; 096,146,196, 896, and 996 were used to define invasive BC cases. Women with BC diagnosed before their first invitation date were excluded.

### Statistical methods

The yearly number of women invited, screened, and recalled for further assessment as well as the participation and recall rates are presented in Table 1. Mode of detection based on the EU guidelines was determined for BC cases by using the individual screening histories and outcome of screening and categorized into screen-detected case at the prevalent and the subsequent screening rounds, interval cancer (IC), and non-participant (NP) [10]. Individual person-years were calculated from the date of first invitation to the date of BC diagnosis, two years after the last invitation or December 31, 2014, whichever came first. The IC ratio was calculated as the number of ICs divided by the number of prevalent screen-detected cases (PSDs), subsequent screen-detected case (SSDs), and ICs. The age-specific BC incidence rate is reported in four age groups: 50–54, 55–59, 60–64, and 65–69 (Table 2). For women who were invited to screening after age 69, the person-years and number of breast cases were included in the 65–69 age group.

**Table 1:** Number of women invited and screened, participation rate, number of women recalled for further assessment, and recall rate by year of invitation in Stockholm breast cancer screening

Year	Women invited	Women screened	Participation rate (%)	Women recalled	Recall rate (%)
1989	32,401	23,133	71.4	637	2.75
1990	77,740	56,488	72.7	1736	3.07
1991	84,267	58,426	69.3	1448	2.48
1992	74,665	56,090	75.1	1239	2.21
1993	78,040	58,132	74.5	1279	2.20
1994	83,156	61,285	73.7	1371	2.24
1995	82,260	59,078	71.8	1275	2.16
1996	84,562	62,809	74.3	1385	2.21
1997	87,438	65,069	74.4	1614	2.48
1998	91,229	68,682	75.3	1657	2.41
1999	93,754	63,080	67.3	1587	2.52
2000	83,434	59,840	71.7	1615	2.70
2001	95,640	65,633	68.6	1814	2.76
2002	94,055	67,457	71.7	1852	2.75
2003	87,530	61,430	70.2	1808	2.94
2004	99,012	70,921	71.6	2341	3.30
2005	99,433	70,759	71.2	1998	2.82
2006	95,875	69,931	72.9	1762	2.52
2007	96,134	69,973	72.8	1797	2.57
2008	102,561	75,930	74.0	1717	2.26
2009	110,306	79,953	72.5	1612	2.02
2010	96,693	70,204	72.6	1504	2.14
2011	95,281	69,391	72.8	1465	2.11
2012	109,052	81,949	75.1	1799	2.20
2013	104,149	78,349	75.2	1852*	2.36*
2014	94,486	71,880	76.1	1857*	2.58*
Total	2,333,153	1,695,872	72.7	42021*	2.48*

*Owing to insufficient follow-up time, value is not complete

**Table 2:** Number of person-years, invasive breast cancer cases by detection mode, interval cancer ratio, and breast cancer incidence by age group

Age at invitation	Person-years	PSD	SSD	IC	NP	IC ratio (%)^a^	BCI (per 10^5^)^b^
50–54	1,163,586.1	875	721	736	697	31.6	260
55–59	1,264,796.0	277	1507	955	793	34.9	279
60–64	1,098,407.1	316	2001	872	655	27.3	350
65–69^c^	1,157,223.7	331	2277	975	660	27.2	367
Total	4,684,012.9	1799	6506	3538	2805	29.9	313

*Abbreviations*: *BCI* breast cancer incidence, *IC* interval cancer, *NP* non-participant, *PSD* prevalent screen-detected cases, *SSD* subsequent screen-detected cases

^a^Number of ICs divided by the sum of screen-detected and ICs

^b^Total number of breast cancers divided by person-years

^c^9.65% of the women were also invited to screening at age 70–74 from 2012

#### The four-state Markov model

**Fig. 1:**
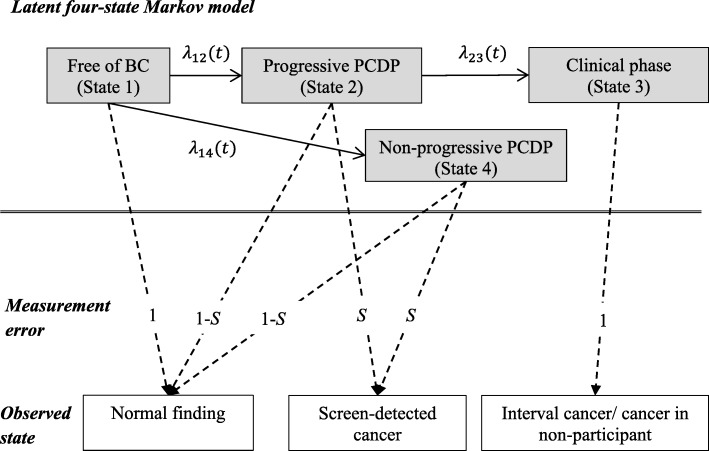
The latent four-state Markov model and the observed states. The possible latent state transition is denoted by arrows, and the probability of being detected in the observed state is denoted by dashed arrows. Abbreviations: *BC* breast cancer, *PCDP* preclinical screen-detectable phase, *S* sensitivity, *λ*_12_(*t*) the transition rate from state 1 to state 2 at time *t*, *λ*_23_(*t*) the transition rate from state 2 to state 3 at time *t*, *λ*_14_(*t*) the transition rate from state 1 to state 4 at time *t*

To model the data collected from the screening program, a hidden Markov model with four latent states and three observed states was used (Fig. 1, redrawn and modified from the original in [8]). Let *X*(*t*) denote the underlying disease process which is unobserved or hidden and is assumed to follow the Markov property, which means that the future status depends only on the current status and is independent of all states before. The four latent states are (1) free of BC, (2) progressive PCDP, (3) CP, and (4) non-progressive PCDP. If the progression of BC is an irreversible procedure, the transition rate (**Λ**(*t*)) at time *t* is as follows.

$$ {\displaystyle \begin{array}{l}\kern14.5em \mathrm{to}\kern2em \\ {}\kern2.25em \mathrm{from}\kern4.1em 1\kern5.6em 2\kern3.5em 3\kern2.9em 4\\ {}\boldsymbol{\Lambda} (t)=\begin{array}{c}1\\ {}2\\ {}3\\ {}4\end{array}\left(\begin{array}{cccc}-{\lambda}_{12}(t)-{\lambda}_{14}(t)& {\lambda}_{12}(t)& 0& {\lambda}_{14}(t)\\ {}0& -{\lambda}_{23}(t)& {\lambda}_{23}(t)& 0\\ {}0& 0& 0& 0\\ {}0& 0& 0& 0\end{array}\right),\end{array}} $$

The transition rate from state *i* to state *j* at age *t* is defined by

$$ {\lambda}_{ij}(t)=\underset{\delta t\to 0}{\lim}\Pr \left(X\left(t+\delta t\right)=j|X(t)=i\right)/\delta t. $$

The transition probability from state *i* to state *j* is defined by *P*_*ij*_(*s*, *t*) = Pr(*X*(*t*) = *j*| *X*(*s*) = *i*) for 0 ≤ *s* ≤ *t*.

We assumed that initiation time (*t*_0_) of the disease process is age 40. It implied that women who participated in the screening program were assumed to be free of BC before age 40. The transition rates were assumed to be constant within intervals, and the transition rate matrix could be expressed by **Λ**(*t*) = **Λ**^*l*^ with entries ${\lambda}_{ij}(t)={\lambda}_{ij}^{(l)}$. In our model, three age intervals, [40, 50),[50, 60), and [60, ∞), which were denoted by *l* = 1, 2, and 3, were defined. In addition, we reparametrized the transition rate from state 1 to state 4 by assuming that the transition rate from state 1 to state 4 was proportional to the rate to state 2 over time. The ratio of two transition rates was represented by $r=\frac{\lambda_{14}(t)}{\lambda_{12}(t)}$ .

#### The observed states and measurement error

The individual screening history, including invitation and participation, combined with outcome of screening indicated the subject’s observed disease states in the screening period. The information was described by three observed states denoted by *Y*(*t*), including (1) negative finding, (2) screen-detected case, and (3) clinical case. The observed states depend on not only the true disease states but also on the accuracy of the screening program. A preclinical BC case might be misclassified as a negative finding (false-negative case) of mammography. The probability of detecting a progressive or a non-progressive PCDP case was defined as test sensitivity (*S*). In contrast, a subject free of BC may be misclassified as an abnormal result of mammography (false-positive case). However, as further diagnostic examinations were performed to confirm the diagnosis, the probability of being misclassified as a cancer case,Pr(*Y*(*t*) = 2| *X*(*t*) = 1), was set to zero. We assumed that the cases in the CP will seek medical care and thus can be identified in the cancer register. The misclassification matrix (***E***) was defined as follows:

$$ {\displaystyle \begin{array}{l}\kern5.5em Y(t)\\ {}\kern1.25em X(t)\kern1.3em 1\kern1.5em 2\kern1.1em 3\\ {}E=\begin{array}{c}1\\ {}2\\ {}3\\ {}4\end{array}\left(\begin{array}{ccc}1& 0& 0\\ {}1-S& S& 0\\ {}0& 0& 1\\ {}1-S& S& 0\end{array}\right)\end{array}} $$

We assumed the misclassification matrix to be constant over age and the test sensitivity of progressive and non-progressive PCDP cases to be the same due to non-differential pathological findings (that is, Pr(*Y*(*t*) = 2| *X*(*t*) = 2) = Pr(*Y*(*t*) = 2| *X*(*t*) = 4) = *S* ). In addition, a false-negative case was assumed to be detected in the next screening round (if not detected clinically before that) to simplify the likelihood function [8].

#### Maximum likelihood estimation

The individual observed states identified from the screening histories combined with the outcomes of screening were used to construct the likelihood function through the transition probabilities. The transition probabilities can be derived from the transition rates by solving the forward Kolmogorov equation [11]. The likelihood function was similar to that used by Wu et al. [8]. In brief, the likelihood contribution of a sequence of observations on an individual subject can be represented by transition probabilities and misclassification probabilities according to the observed states [12]. Since individual screening histories were collected from age 50, the transition rates before age 50 were intractable. The transition rate from state 1 to state 2 for time interval [40, 50) was obtained from the average age-specific incidence in 1989–2004 in Stockholm as reported in the cancer register [13]. Because the closed-form solutions of parameters did not exist, a numerical procedure was required to estimate the parameters and maximize the likelihood. The quasi-Newton (Broyden–Fletcher–Goldfarb–Shanno) and Nelder–Mead methods were used to find the maximum likelihood estimates (MLEs) using the package *optimx* in R software [14]. We applied the Karush–Kuhn–Tucker conditions to determine whether the −2*log likelihood indeed converged [15]. The standard errors of the estimates of parameters were obtained from the inverse of the Hessian matrix of the maximized log-likelihood function. A homogeneous Markov model based on constant transition rates was also estimated, and likelihood ratio test was used to compare the homogeneous and non-homogeneous models. All calculations were performed using the R statistical software.

To check whether the model fitted the data, the observed and expected cumulative incidence curves in the ever-attenders were plotted. The expected number of BC cases was calculated on the basis of the individual screening histories and MLEs of parameters. The annual observed incidence rate was first calculated and the expected number of BC cases in each year was calculated by summing the probability that an individual was in PCDP or CP given her previous states over all at-risk subjects. Follow-up of clinically detected cases was continued until the next supposed examination time (two years after latest scheduled time) [16].

#### Estimation of frequency of overdiagnosis

There are several different definitions of overdiagnosis in the literature because of the choice of denominator [17]. In the present study, overdiagnosis due to screen detection of non-progressive cancer was estimated. We used the number of screen-detected cases as the denominator and the expected number of detected non-progressive BCs as the numerator. Here, the expected number was calculated as the number of screen-detected cases at each screening round multiplied by the estimated probability that a detected BC would be non-progressive. The estimated probability was calculated as follows:

$$ {\displaystyle \begin{array}{l}\frac{\Pr \left(\mathrm{a}\kern0.28em \mathrm{non}-\mathrm{progressive}\kern0.28em \mathrm{BC}\kern0.28em \mathrm{is}\kern0.34em \mathrm{delected}\kern0.28em \mathrm{at}\kern0.28em {t}_k\right)}{\Pr \left(\mathrm{a}\kern0.28em \mathrm{non}-\mathrm{progressive}\kern0.28em \mathrm{BC}\kern0.28em \mathrm{is}\kern0.34em \mathrm{delected}\kern0.28em \mathrm{at}\kern0.28em {t}_k\right)+\Pr \left(\mathrm{a}\kern0.28em \mathrm{progressive}\kern0.28em \mathrm{BC}\kern0.28em \mathrm{is}\kern0.34em \mathrm{delected}\kern0.28em \mathrm{at}\kern0.28em {t}_k\right)}\\ {}=\frac{\left({A}_1+{A}_2\right)}{\left({A}_1+{B}_1\right)+\left({A}_2+{B}_2\right)}\\ {}=\frac{{\hat{P}}_{11}\left({t}_{k-2},{t}_{k-1}\right)\times {\hat{P}}_{14}\left({t}_{k-1},{t}_k\right)\times \hat{S}+{\hat{P}}_{14}\left({t}_{k-2},{t}_{k-1}\right)\times \left(1-\hat{S}\right)}{\left\{{\hat{P}}_{11}\left({t}_{k-2},{t}_{k-1}\right)\times \left({\hat{P}}_{14}\left({t}_{k-1},{t}_k\right)+{\hat{P}}_{12}\left({t}_{k-1},{t}_k\right)\right)\times \hat{S}+\left({\hat{P}}_{14}\left({t}_{k-2},{t}_{k-1}\right)+{\hat{P}}_{12}\left({t}_{k-2},{t}_{k-1}\right)\times {\hat{P}}_{22}\left({t}_{k-1},{t}_k\right)\right)\times \left(1-\hat{S}\right)\right\}\operatorname{}}\end{array}} $$

Here, *k* denotes the screening round and the corresponding age is *t*_*k*_ ${\widehat{P}}_{ij}\left({t}_{k-1},{t}_k\right)$ represents the estimated transition probability from state *i* at time *t*_*k* − 1_ to state *j* at time *t*_*k*_, and$\widehat{S}$denotes the estimated test sensitivity. The first part of the denominator represents the probability of a subject who stays in state 1 before *t*_*k* − 1_, transits to either state 4 (*A*_1_) or state 2 (*B*_1_) between *t*_*k* − 1_ and *t*_*k*_, and then is detected by screening. The second part of the denominator represents the probability of a subject who transits to state 4 (*A*_2_) or 2 (*B*_2_) between *t*_*k* − 2_and *t*_*k* − 1_ but is not detected at time *t*_*k* − 1_ and stays in same state at time *t*_*k*_. Because the probability for staying in state 4 is one, it is omitted from the equation. The numerator represents the probability of a subject who transits from state 1 to state 4 between either time *t*_*k* − 1_ and *t*_*k*_ (*A*_1_) or *t*_*k* − 2_ and *t*_*k* − 1_(*A*_2_).

The 95% confidence interval (CI) of frequency of overdiagnosis was estimated by simulating the variation in the estimation of overdiagnosis. We drew the values from the multivariate normal distribution (with mean vectors equal to the MLEs of parameters and the covariance matrix equal to the estimated covariance matrix) 1000 times and computed the expected number of non-progressive BCs for each drawn value to reflect the sample variation of overdiagnosis [18].

## Results

During the study period 1989–2014, 2,333,153 invitations were sent and 1,695,872 women participated in the screening program (Table 1). The average participation rate was 72.7%. The yearly recall rate varied between 2.02% and 3.30% (median 2.48%).

The incidence rates of invasive BC in women 50–54, 55–59, 60–64, and 65–69 years old were 260, 279, 350, and 367 per 100,000 women, respectively (Table 2). Almost half (48.6%) of the PSDs were found in women 50–54. The IC ratio was higher in women 50–59 (33.3%) than in women 60 or older (27.3%).

**Fig 2:**
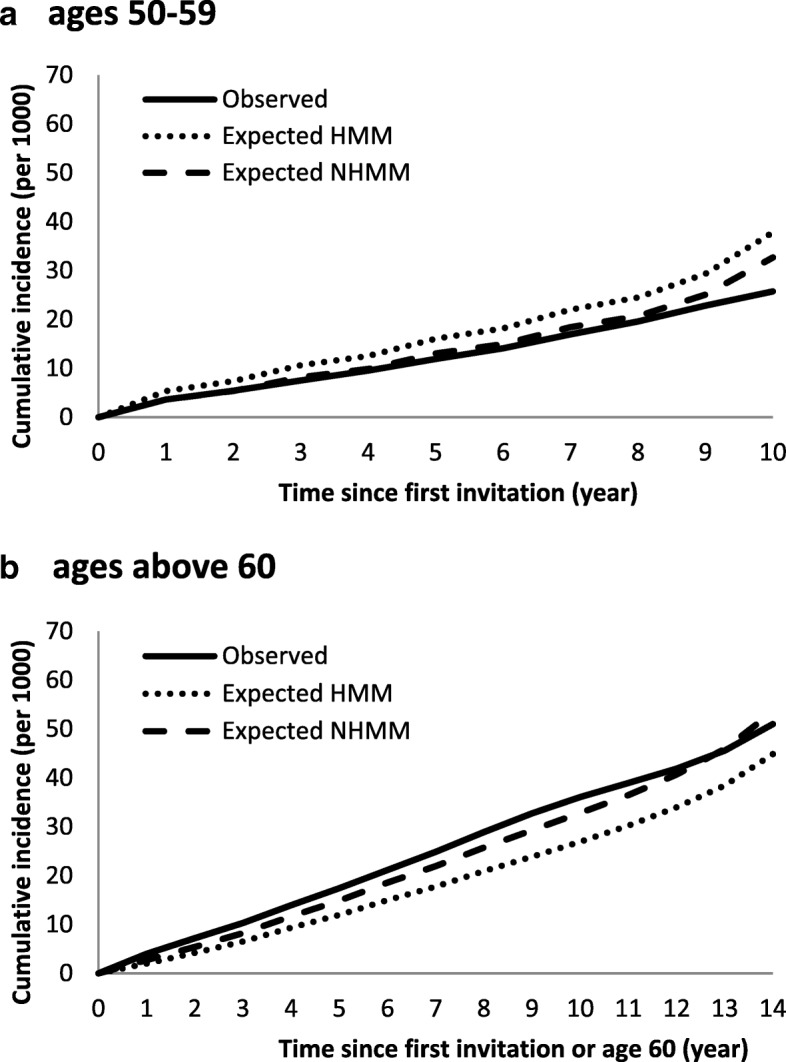
The observed and expected cumulative incidence rate in the homogeneous (HMM) and the non-homogeneous multi-state (NHMM) models. (a) Ages 50–59. (b) Ages above 60

The transition rates from free of BC to progressive PCDP were 276 and 381 per 100,000 women-years for women 50–59 and 60–69, respectively (Table 3). The mean sojourn times (MSTs) in age 40–49, 50–59, and 60–69 were 2.60 (95% CI 2.31–2.89), 2.16 (95% CI 2.03–2.29), and 3.52 (95% CI 3.31–3.73) years, respectively. The ratio of *λ*_14_(*t*) to*λ*_12_(*t*) was 0.00182 (95% CI 0–0.00523), and the test sensitivity was 88% (95% CI 85.2%–90.9%). The likelihood ratio test showed that the non-homogeneous model fits the data better than the homogeneous model (chi-squared = 854 with 3 degrees of freedom, *P* <0.001), which is visually confirmed by the cumulative observed and expected incidence curves (Fig. 2). The results showed that the homogeneous model overestimated the risk of BC in the ages of 50–59 and underestimated the risk in the ages above 60. The expected cumulative incidence curve in the non-homogeneous model was close to the observed incidence curve, indicating that the model fit was adequate. Table 4 shows the estimation of overdiagnosis from non-progressive detected BC cases. Among the 8305 screen-detected invasive cases, the expected number of non-progressive BC cases detected by screening was 35.9, which corresponds to 0.43% (95% CI 0.10%–2.18%) overdiagnosis. There were 15.6 (0.87%, 95% CI 0.20%–4.31%) and 20.3 (0.31%, 95% CI 0.07%–1.59%) estimated non-progressive BC cases in the prevalent and subsequent rounds of screening, respectively.

**Table 3:** Maximum-likelihood estimates and 95% confidence intervals based on the non-homogeneous and the homogeneous multi-state model

Description	Parameter	MLE (95% CI)			
		Non-homogeneous model			Homogeneous model
		40–49 years	50–59 years	60–69 years^a^	
Transition rate from free of BC to progressive PCDP	*λ*_12_(*t*)	–	0.00276 (0.00269, 0.00283)	0.00381 (0.00371, 0.00390)	0.00306 (0.00301, 0.00311)
Transition rate from progressive PCDP to CP	*λ*_23_(*t*)	0.385 (0.342, 0.428)	0.464 (0.436, 0.492)	0.284 (0.267, 0.301)	0.418 (0.400, 0.436)
Ratio of *λ*_14_(*t*) to *λ*_12_(*t*)	*r*	0.00182 (0, 0.00523)			9.999×10^−4^ (0, 3.432×10^−3^)
Sensitivity	*S*	0.880 (0.852, 0.909)			0.924 (0.901, 0.947)
-2*log(likelihood)	NA	198,024			198,878
Mean sojourn time (year)	$\frac{1}{\lambda_{23}(t)}$	2.60 (2.31, 2.89)	2.16 (2.03, 2.29)	3.52 (3.31, 3.73)	2.39 (2.29, 2.49)

*Abbreviations*: *BC* breast cancer, *CI* confidence interval, *CP* clinical phase, *MLE* maximum likelihood estimate, *MST* mean sojourn time, *NA* not applicable, *PCDP* preclinical screen-detectable phase

Likelihood ratio test of the non-homogeneous versus the homogeneous model: ${\chi}_{(3)}^2$ = 854, *P* <0.001

^a^9.65% of the women were invited to screening at age 70–74

**Table 4:** Number of screen-detected cases, expected number of detected non-progressive breast cancers, and the frequency of overdiagnosis (percentage) by round of screening

Screening round	Number of screen-detected cases	Expected number of detected NPBCs	Overdiagnosis (95% CI)
Prevalent	1799	15.57	0.87% (0.20%, 4.31%)
Subsequent	6506	20.33	0.31% (0.07%, 1.59%)
Overall	8305	35.90	0.43% (0.10%, 2.18%)

*Abbreviations*: *CI* confidence interval, *NPBC* non-progressive breast cancer

## Discussion

We used a non-homogeneous multi-state model to estimate the frequency of overdiagnosis from the expected number of non-progressive BCs among the screen-detected cancer cases in the population-based organized screening program with mammography in women 50–69 in Stockholm County, Sweden. We found that only 0.43% of the screen-detected invasive cases were overdiagnosed. The frequency of overdiagnosis in the prevalent round was three times higher than that in the subsequent rounds. We showed that the non-homogeneous model fitted the data better than the homogeneous model.

Published estimates of the frequency of overdiagnosis have varied because of type of screening program, study design, choice of control group, estimation method, and adjustment for lead time (the time by which screening advances the diagnosis compared with absence of screening) [2, 19–22]. Overdiagnosis based on Swedish data has been estimated in four RCTs and two observational studies.

### Estimation of overdiagnosis based on the Swedish RCTs

In the Stockholm trial, two screening rounds were performed for 40,318 women 40 to 64 years old, and 20,000 controls were also invited to one screening round at the end of the trial. Two estimates of overdiagnosis were made on the basis of the Stockholm trial. Gotzsche found 49% overdiagnosis by comparing the relative risk of all BCs in the screening period [23]. However, failing to separate the increasing incidence due to earlier detection of cancer (the so-called lead-time problem) results in overestimation of overdiagnosis. Moss found that the invasive (0.81 versus 0.85 per 1000 person-years in the screened and in the control groups) and all BC cumulative incidence rates (0.88 versus 0.91 per 1000 person-years in the screened and the control groups) were similar between two arms over 15 years follow-up [24]. It should be noted that because the control group was also invited to a single screen, which might lead to some overdiagnosis in the control group, overdiagnosis probably was underestimated in this approach. Our findings of the frequency of overdiagnosis in the subsequent screening rounds of the organized screening program were consistent with Moss’s finding in the Stockholm trial that showed no evidence of overdiagnosis as a result of incident screens [25].

Similar results were found in the Two-County trial and the Gothenburg trial; −0.02 and −0.03 per 1000 absolute excess cumulative incidences of all BCs were found in the screened group in the two trials, respectively [24]. Duffy et al. applied a homogeneous four-state model to quantify the overdiagnosis [4]. The frequency of overdiagnosis in the prevalent and the two subsequent rounds were 3.1%/4.2% and 0.3%/0.3%, respectively, in the Two-County/Gothenburg trials. These estimates are in line with our estimates and confirm a low level of overdiagnosis. Using 29-year follow-up data from one county of the Two-County trial, Yen et al. further confirmed that screening did not lead to excess incidence of BC in the screened group (risk ratio 1.00) where 100,000 additional screens were performed compared with the control group. No evidence of overdiagnosis for invasive or *in situ* BC was found [26].

The estimate for overdiagnosis based on the Malmö I trial has been considered reliable because of the stop-screen design (women in the control group were never invited to screening) and an adequate follow-up time [27, 28]. Zackrisson et al. estimated the frequency of overdiagnosis at 10% for all BCs and 7% for invasive BCs in women 55 to 69 at random assignment by comparing the incidence rate between the invited and control groups at 15 years of follow-up, after the end of the trial [28]. The United Kingdom BC panel recalculated the estimated overdiagnosis by comparing the excess numbers with different denominators, such as the number of cancers diagnosed over the whole follow-up period in the control group/invited group or the number of cancers/screen-detected cancers diagnosed in the screening period in the invited group [27]. The estimates varied from 11% to 29%. Although RCTs may provide a good opportunity to quantify overdiagnosis, the generalizability for the current organized screening program remains dubious.

### Estimation of overdiagnosis based on the organized screening programs

Zahl et al. used the age-specific incidence of invasive BC during 1971–2000 to quantify the increasing incidence after introduction of mammographic screening in Sweden [29]. They estimated that the frequency of overdiagnosis in women 50–69 years old was 45%; however, lead time was not properly adjusted for and the increase in the incidence over time was not considered. Jonsson et al. also applied the incidence rate approach to quantify overdiagnosis in 11 out of 20 countries after implementing organized screening [30]. The pre-screening incidence (15 years before the start of screening) was used to calculate the expected incidence in the absence of screening during the screening period until year 2000. In the stable phase, overdiagnosis rates were estimated at 54% and 21% for the 50–59 and 60–69 age groups, respectively, after lead-time adjustment. It should be noted that the increased incidence might result from a prevalent screening effect among newcomers, potential changes in risk factors leading to changing trends, and so on; therefore, it should not be attributed entirely to overdiagnosis [30]. However, the data from the organized screening program in Stockholm County were not included in this study and the choice of pre-screening period might have influenced on the estimation of overdiagnosis [3]. Therefore, it was difficult to compare with our findings.

### Estimation of mean sojourn time

Our estimates of the MSTs for women ages 40–49, 50–59, and 60–69 (2.60, 2.16, and 3.52 years) were lower than previously reported MSTs in the Two-County trial (2.44, 3.70, and 4.17 years) [31]. There are several reasons for the shorter MSTs in our study. First, the sojourn time in our model represents the sojourn time in the progressive BCs. The non-progressive BCs having infinite sojourn time were separated. Second, it has been shown that there is an association between hormone replacement therapy (HRT), BC risk, and sojourn time [32, 33]. In Sweden, the use of HRT increased starting in 1990 and decreased after 2002 and the majority of HRT use was in the 50–59 age group [34]. HRT use increases the risk of invasive lobular carcinoma, which has a shorter sojourn time than ductal carcinoma [35]. This might explain why we got lower estimates of MST in women 50–59 years old. Another explanation can be that Duffy et al. used age at random assignment to classify the population into age groups regardless of how old they were at the end of the study and the MST was estimated separately for these groups. For example, women 50–59 at random assignment were 57–66 years old at the end of the trial and thus were an average of 3–4 years older during the study period. The estimate of longer MST in 50–59 age group found by Duffy et al. may be partially attributable to the longer MST observed at ages 60–69. In contrast, in our model, a woman might contribute to the likelihood for estimation of MST in different age intervals as they move through the age groups.

### Concerns about the *in situ* cancers

The incidence of ductal carcinoma *in situ* cancer has increased significantly since the introduction of the organized screening program [36]. This increase has been considered to be a marker of overdiagnosis [37]. A sensitivity analysis was performed by combining the *in situ* and invasive BCs in the same state to estimate overdiagnosis (Additional files 1 and 2). There were 51.6 non-progressive detected BC cases found among 9631 *in situ* and invasive BC cases, corresponding to 0.54% overdiagnosis (Additional file 3). Although slightly higher overdiagnosis was found, the frequency of overdiagnosis was still low. Similar findings were demonstrated by the six-state model in women 40–49, suggesting that the majority of screen-detected *in situ* cancers would have presented clinically in the absence of screening [7, 38].

### Strengths and limitations

Our estimate of overdiagnosis based on a non-homogeneous model and large-scale screening data has several strengths. First, to the best of our knowledge, this is the first study using individual screening histories to quantify the frequency of overdiagnosis in the Stockholm organized screening program. Aggregated data used in most studies cannot reflect the actual exposure of screening. In the Stockholm screening program, individual screening histories were collected from the start, quality-checked, and regularly stored in the register. Date of and status of participation, mammographic results, follow-up assessments, and cancer outcomes were prospectively recorded [9, 39]. Second, the unscreened population (expected number of BCs in the absence of screening) was obtained from natural history modeling that provides the same characteristics (risks) between the unscreened population and screened population. Bias, which results from the choice of control groups, could thus be prevented. Third, age-specific incidence and sojourn time were taken into account in our model. Piecewise constant transition rates were used in the non-homogeneous model and fitted the data better than the traditional homogeneous model with constant rates.

There are some limitations which may have influenced our estimates of overdiagnosis. First, the detection mode of BC cases might be misclassified. For example, women born in 1920–1941 might have been invited to at least one screening round in the Stockholm trial. The SSDs might be misclassified as PSDs. Lidbrink et al. found that, in the organized screening in Stockholm, the tumor size in the screening units performing prevalent screening was similar to that in the unit where the trial was conducted [39]. Mammography performed outside of the organized screening program might also bias our results. The participation rate in the more densely populated counties in Sweden, including Stockholm County, has been lower than in other counties in Sweden, which might be due to higher access to private mammography, in particular during the first years of the program [1, 40]. The cancer cases diagnosed in the private sector might be misclassified as NPs in the organized screening program. The risk of progressing to the CP might be overestimated, leading to an underestimation of overdiagnosis.

Second, overdiagnosis resulting from the detection of progressive cancers in women who died before the cancer became symptomatic was not counted in our study. A possible extension of the model is to consider death as a separate state [7, 41]. Besides, in Stockholm, the all-cause mortality rates in women 50–59 and 60–69 from year 1989 to 2014 were 3.2 and 7.98 per 1000 women-years [42]. Thus, during a 2.16-year sojourn time, 6.91 deaths per thousand women might be expected in those progressive screen-detected breast cases at ages 50–59. In other words, a reasonable estimate of overdiagnosis due to death would be approximately 0.69% for women 50–59. The corresponding estimate for the women 60–69 would be 2.8% given a 3.52-year sojourn time. The true value will be lower after considering the difference between lead time and sojourn time.

Third, the assumptions made in modeling of the natural history should be considered. We assumed the test sensitivity to be constant over time, age, and type of BC. Owing to lack of data, the effect of improvement of screening tools, like digital mammography, was not possible to take into account. In addition, false-negative cases were assumed to be detected in the next screening round for simplification of the likelihood function. It might slightly overestimate the test sensitivity. Furthermore, our non-homogeneous model requires a certain initiation time where the true state either is known or can be modeled [12]. We restricted the model so that the risk of BC was zero before the age of 40 years. A sensitivity analysis was performed to check this model assumption. The results were similar when the initiation time was assumed to be 35 or 45 years (not shown). The average incidence rate from the Stockholm cancer register for the years 1989–2004 was used to approximate the transition rate from free of BC to progressive PCDP in the 40–49 age group. The underlying preclinical incidence rate might be higher than the clinical incidence rate since the incidence increases with age. This might affect other estimates of parameters, especially the MST in women 40–49, if the rate does not represent the background incidence.

Fourth, a more robust CI of the estimate of overdiagnosis could be calculated through the bootstrapping method. However, since the individual screening history for over 400,000 women was used for estimation, the estimation procedure was very time-consuming.

Another important issue is that the likelihood function might be flat and lead to an identifiability problem. Even if our model fitted the data well, we cannot exclude a misspecification of the model. The genuine progression of BC could have been oversimplified in our four-state model. Owing to insufficient or incomplete (censored) data, it might be difficult to get the correct estimates. To successfully estimate other parameters and to further quantify the overdiagnosis, our model relies on the information from clinically detected cancers, including ICs and NPs (who were the most informative cases in the dataset because the exact transition time to CP is known). Earlier, we showed that a certain proportion (5–10%) of an unscreened group, like never-attenders, can stabilize the model [8]. Therefore, the inspection of the likelihood function seems to be necessary. We have checked the Karush–Kuhn–Tucker conditions to make sure the optimization algorithm indeed converged.

Our findings provide the following insights for future research. First, it would be valuable to assess the period effects on the BC risk, sojourn time, and sensitivity to investigate how exposure to HRT and digital mammography have influenced the frequency of overdiagnosis. Second, further comparison of the overdiagnosis estimates based on other evaluation methods, like the cumulative incidence method, in the same dataset needs to be carried out to provide more solid evidence for policy makers to further confirm the findings [21].

The frequency of overdiagnosis in the organized screening program depends on the latent proportion of non-progressive BC cases but also on the screening program, where a higher participation rate or improved sensitivity due to better screening instruments will lead to higher frequency of overdiagnosis. The balance between benefit and harm of screening should be considered and thus regularly monitoring the mortality reduction and overdiagnosis will be necessary [43].

## Conclusions

Our findings suggest that the overdiagnosis in the population-based organized biennial mammographic screening for women 50–69 in Stockholm County, Sweden, is a minor phenomenon. The proportion of frequency of overdiagnosis in the prevalent screening round was higher than that in subsequent rounds but still low. The non-homogeneous model fitted the data better than the simpler, traditional homogeneous model.

## Additional files

Additional file 1: **Table S1.** Number of person-years, *in situ* and invasive breast cancer cases by detection mode, interval cancer ratio, and breast cancer incidence. (DOCX 20 kb)

Additional file 2: **Table S2.** Maximum-likelihood estimates (MLEs) and 95% confidence intervals (CIs) based on the non-homogeneous and the homogeneous multi-state model of the *in situ* and invasive breast cancer. (DOCX 29 kb)

Additional file 3: **Table S3.** Number of screen-detected cases, expected number of detected non-progressive *in situ* combined with invasive breast cancers (non-progressive breast cancer, or NPBC), and the frequency of overdiagnosis (percentage) by round of screening. (DOCX 13 kb)

## Abbreviations

BC: Breast cancer
CI: Confidence interval
CP: Clinical phase
HRT: Hormone replacement therapy
IC: Interval cancer
MLE: Maximum likelihood estimate
MST: Mean sojourn time
NP: Non-participant
PCDP: Preclinical screen-detectable phase
PSD: Prevalent screen-detected case
RCT: Randomized controlled trial
SSD: Subsequent screen-detected case
