# Supplementary material for: Overdiagnosis in the population-based organized breast cancer screening program estimated by a non-homogeneous multi-state model: a cohort study using individual data with long-term follow-up
Source: Breast Cancer Res. 2018 Dec 17;20:153. doi: 10.1186/s13058-018-1082-z (PMC6296133; doi:10.1186/s13058-018-1082-z)
Supplement: Supplementary file 1 — Table S1. Number of person-years, in situ and invasive breast cancer cases by detection mode, interval cancer ratio, and breast cancer incidence. (DOCX 20 kb) [file 13058_2018_1082_MOESM1_ESM.docx]

**Additional file 1:**

Table S1. Number of person-years, in situ and invasive breast cancer cases by detection mode, interval cancer ratio, and breast cancer incidence.

| Age at invitation | Person-years | PSD | SSD | IC | NA | ICR (%)^a^ | BC Incidence  (per 10^5^)^b^ |
| --- | --- | --- | --- | --- | --- | --- | --- |
| 50-54 | 1163586.1 | 1092 | 888 | 796 | 754 | 28. 7 | 303.4 |
| 55-59 | 1264796.0 | 320 | 1757 | 1009 | 853 | 32.7 | 311.4 |
| 60-64 | 1098407.1 | 347 | 2285 | 927 | 704 | 26.0 | 388.1 |
| 65-69^c^ | 1157223.7 | 382 | 2560 | 1041 | 707 | 26.1 | 405.3 |
| Total | 4684012.9 | 2141 | 7490 | 3773 | 3018 | 28.2 | 350.6 |

PSD: prevalent screen-detected cases, SSD: subsequent screen-detected cases, IC: interval cancer, NP: non-participant, ICR: interval cancer ratio

^a^ number of interval cancers divided by the sum of screen detected and interval cancers

^b^ total number of breast cancers divided by person-years

^c^ 9.65% of the women were also invited to screening in age 70-74 from 2012
