# Supplementary material for: Overdiagnosis in the population-based organized breast cancer screening program estimated by a non-homogeneous multi-state model: a cohort study using individual data with long-term follow-up
Source: Breast Cancer Res. 2018 Dec 17;20:153. doi: 10.1186/s13058-018-1082-z (PMC6296133; doi:10.1186/s13058-018-1082-z)
Supplement: Supplementary file 2 — Table S2. Maximum-likelihood estimates (MLEs) and 95% confidence intervals (CIs) based on the non-homogeneous and the homogeneous multi-state model of the in situ and invasive breast cancer. (DOCX 29 kb) [file 13058_2018_1082_MOESM2_ESM.docx]

**Additional file 2:**

Table S2. Maximum-likelihood estimates (MLE) and 95% confidence intervals (CIs) based on the non-homogeneous and the homogeneous multi-state model of the in situ and invasive breast cancer

| Description | Parameter | MLE (95% CI) | | | | |
| --- | --- | --- | --- | --- | --- | --- |
|  |  | Non-homogeneous model | | | | Homogeneous model |
|  |  | 40-49 y | | 50-59 y | 60-69 y^a^ |  |
| Transition rate from free of BC to progressive PCDP | $\lambda_{12}(t)$ | - | | 0.00308  (0.00300, 0.00316) | 0.00420  (0.00410, 0.00429) | 0.00336  (0.00331, 0.00342) |
| Transition rate from progressive PCDP to CP | $\lambda_{23}(t)$ | 0.288  (0.256, 0.320) | | 0.434  (0.408, 0.458) | 0.271  (0.256, 0.287) | 0.416  (0.400,0.433) |
| Ratio of $\lambda_{14}(t)$ to $\lambda_{12}(t)$ | $r$ | 0.00241  (0, 0.00621) | | | | 1.768$\times10^{-4}$  (0, 8.498$\times10^{-4}$) |
| Sensitivity | $S$ | 0.883  (0.857, 0.910) | | | | 0.930  (0.908, 0.952) |
| -2*log(likelihood) | NA | 217619 | | | | 215142 |
| Mean sojourn time (year) | $\frac{1}{\lambda_{23}(t)}$ | 3.47 | 2.31 | | 3.69 | 2.40 |

PCDP: preclinical screen-detectable phase, CP: clinical phase, MLE: maximum likelihood estimate, CI: confidence interval, MST: mean sojourn time, NA: not applicable

Likelihood-ratio test of the non-homogeneous vs the homogeneous model : $\chi_{(3)}^{2}$=2477, p-value<0.001

^a^ 9.65% of the women were invited to screening in age 70-74
