# Supplementary material for: Overdiagnosis in the population-based organized breast cancer screening program estimated by a non-homogeneous multi-state model: a cohort study using individual data with long-term follow-up
Source: Breast Cancer Res. 2018 Dec 17;20:153. doi: 10.1186/s13058-018-1082-z (PMC6296133; doi:10.1186/s13058-018-1082-z)
Supplement: Supplementary file 3 — Table S3. Number of screen-detected cases, expected number of detected non-progressive in situ combined with invasive breast cancers (non-progressive breast cancer, or NPBC), and the frequency of overdiagnosis (percentage) by round of screening. (DOCX 13 kb) [file 13058_2018_1082_MOESM3_ESM.docx]

**Additional file 3:**

Table S3. Number of screen-detected cases, expected number of detected non-progressive in situ combined with invasive breast cancers (NPBC) and the frequency of overdiagnosis (%) by round of screening.

| Screening round | No. of screen-detected cases | Expected number of detected NPBC | Overdiagnosis (%) |
| --- | --- | --- | --- |
| Prevalent | 2141 | 21.5 | 1.01% |
| Subsequent | 7490 | 30.1 | 0.41% |
| Overall | 9631 | 51.6 | 0.54% |

NPBC: non-progressive breast cancer
